# The Motor Recovery Related with Brain Lesion in Patients with Intracranial Hemorrhage

**DOI:** 10.1155/2015/258161

**Published:** 2015-03-31

**Authors:** Kyung Bo Lee, Joon Sung Kim, Bo Young Hong, Young Dong Kim, Byong Yong Hwang, Seong Hoon Lim

**Affiliations:** ^1^Department of Rehabilitation Medicine, St. Vincent's Hospital, College of Medicine, The Catholic University of Korea, 93 Jungbu-daero, Paldal-gu, Suwon 442-723, Republic of Korea; ^2^Human Movement Research Center, Daejeon 302-852, Republic of Korea; ^3^Department of Physical Therapy, Yongin University, Yongin 449-714, Republic of Korea

## Abstract

Although studies have demonstrated that several specific brain lesions are related to the severity of functional outcomes, the effects of specific brain lesions are not yet clear. This study investigated the effects of hemorrhagic stroke lesions on motor recovery. Eleven subjects with hemorrhagic stroke were assessed. Using the Fugl-Meyer Assessment and functional ambulation category, clinical motor and sensory impairments were tested four times in total: initially within 2 weeks and 1, 3, and 6 months after the onset of stroke. Brain lesions and size were evaluated using MRIcron, SPM8, and Talairach Daemon software. Trunk control, motor function in the lower limbs, and sensory function improved significantly within 3 months, after which the change was no longer significant. Upper limb function and gait were unchanged within 1 month but improved significantly 3 months after onset and continued to improve for 6 months. Involvement of the anterior putamen, internal capsule, thalamus, periventricular white matter, and premotor cortex was related to poor upper limb recovery in patients with hemorrhagic stroke. These results should be useful for planning rehabilitation strategies and understanding the prognosis of hemorrhagic stroke.

## 1. Introduction

The recovery of motor function, including hand function, sitting, and other motor activities of daily living (ADL), is an important outcome in stroke patients [1]. Several studies have investigated the factors influencing motor recovery and the relationships between brain lesions and function [2–4]. In these studies, the size of the lesion in the brain was not related to functional outcome [2, 3]. Few studies have reported on the relationships between brain lesions and functional recovery [5–7].

Previously, we revealed patterns of clinical recovery in subacute stroke patients [8]. We want to investigate the effect of stroke lesion on motor recovery. Although studies have demonstrated the effect of brain lesions on functional outcomes, the effects of specific brain lesions are not yet clear. The primary aim of this study was to investigate the effect of stroke lesion on motor recovery in patients with hemorrhagic stroke. In addition, this study evaluated the recovery pattern of functional ability.

## 2. Methods

### 2.1. Study Design and Participants

This was a longitudinal observational clinical trial. Eleven, right handed, subjects with hemorrhagic stroke were recruited from the Department of Rehabilitation Medicine at St. Vincent's Hospital. All of the subjects had suffered supratentorial intracerebral hemorrhages (ICH) and met the following criteria: (1) a first-ever unilateral stroke, (2) ability to follow verbal instructions, (3) inability to walk within 2 weeks after the ICH, and (4) a Fugl-Meyer Assessment (FMA) score lower than 60 for the upper extremity or lower than 28 for the lower extremity [9].

Exclusion criteria were (1) presence of knee joint effusion determined using US, (2) a history of knee injury or surgery or history of knee injection within 3 months, (3) a history of inflammatory arthritis or inflammatory myopathy, (4) diabetes mellitus, or (5) peripheral nervous disease.

From all subjects, we obtained demographic and brain magnetic resonance imaging (MRI) data and evaluated motor and sensory impairments clinically. The brain lesion and its size were evaluated using high-resolution 1.5 T anatomical MRI with a 5 mm slice thickness within 30 days after the ICH. Initially, 15 subjects were recruited, but four did not complete the study due to complications (e.g., hydrocephalus) or loss to follow-up.

All subjects received physical and occupational therapies on the basis of a neurodevelopmental treatment approach (physical therapy) and task orientated approach (occupational therapy). The rehabilitation program of all subjects had started within 1 month after onset (17.6 ± 5.6 days). The treatment continued up to 6 months after onset, consisted of with 1-2 hours per day, 5 days a week, including each of physical and occupational therapies. They also received speech therapy (ST), as needed. The interventions were mainly focused on using and strengthening the affected limb, basic mat activity, symmetric weight bearing and transfer activities, and gait training but were not performed exclusively for a particular purpose.

The study was approved by the Ethics Committee of the Catholic University of Korea. Written consent was obtained from all subjects according to the Declaration of Helsinki.

### 2.2. Clinical Evaluations

Clinical tests were conducted with FMA, Trunk Impairment Scale (TIS), and functional ambulation category (FAC) [8, 10–12]. Motor and sensory function were evaluated using the FMA [10]. In this study, the score reported did not include the coordination subscore (i.e., the highest scores achievable were 60 and 28 for the upper and lower extremities, resp.). Trunk balance was assessed using the TIS [12]. The ability to ambulate was tested using the FAC [11]. All of the clinical data were obtained at the initial assessment (within 2 weeks) and 1, 3, and 6 months after onset. At 6 months after onset, the patients were divided into two groups: (1) poor recovery with upper limb mass synergic movement (FMA arm score ≤ 18) and (2) good recovery with isolated upper limb movement (FMA arm score > 18). We considered an FMA arm score ≥ 18 to indicate isolated movement recovery.

### 2.3. Lesion Tracing and Analysis Procedures

Lesion size was calculated using the Picture Achieved Communication System (PACS, Marotech, Korea), and the absolute lesion size (cm^3^) was determined by multiplying the sum of all lesion areas in each plane by the slice thickness.

Lesion locations were subject to mutual analysis using MRIcron. The origin of the image (0, 0, 0 mm coordinates) was reoriented such that it was located close to the anterior commissure. Volume of interest (VOI) images of each patient were traced using MRIcron (http://www.mricro.com/mricron/). The VOI images were transformed to the left hemisphere. The tracings were coregistered to the Montreal Neurological Institute brain template [13]. To analyze the mutual lesion maps, the following process was used. First, the T1 images were roughly aligned to standard space. This approximately locates the anterior commissure, which will aid in subsequent normalization. Then, the T2 scan was coregistered to the space in the T1 scan. The T2 image is moved and rotated until closely aligned to the T1 image. Third, the T1 image was aligned to stereotaxic space using unified segmentation and normalization in SPM8. To identify relevant anatomic structures implicated in the analyses, Talairach Daemon (http://www.talairach.org/) was used [14].

### 2.4. Statistical Analysis

Statistical analyses were performed using the SPSS software (ver. 11.5; SPSS Inc., Chicago, IL, USA). All tests were two-tailed, and a *P* value <0.05 was deemed to be significant. The Gaussian distribution was evaluated using the Shapiro-Wilk test. Parametric and nonparametric statistics were used to describe recovery after stroke. The changes in the recovery scores were evaluated using the Friedman test. The relationships between clinical recovery and the isolated movement recovery were assessed using the Mann-Whitney test.* Post hoc* analysis was conducted using the Bonferroni correction (*P* < 0.0167; 0.05/3).

## 3. Results

The study enrolled 11 patients (age 47.3 ± 15.1 years; four women, seven men). Six of the patients had left hemiplegia and the remaining five had right hemiplegia. The mean lesion volume was 34.51 ± 18.12 cm^3^ (Table 1).

The recoveries assessed by clinical evaluation with time are shown in Table 2. Trunk control, motor function of the upper and lower limbs, sensory function, and gait improved significantly with time. Trunk control, motor function in the lower limb, and sensory function improved significantly within 3 months, after which they did not change significantly. Upper limb function and gait did not change within 1 month, improved significantly 3 months after onset, and continued to improve for 6 months.

**Figure 1:**
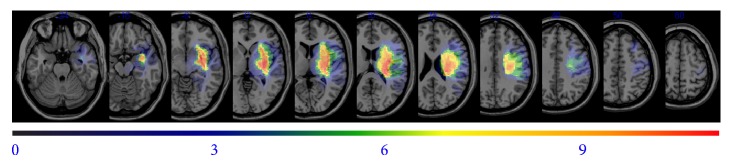
Overlay of lesions for patients with hemorrhagic stroke. Voxels damaged in 1 patient are shown in purple and shades toward the red end of spectrum denote voxels where larger numbers of patients were lesioned.

**Figure 2:**
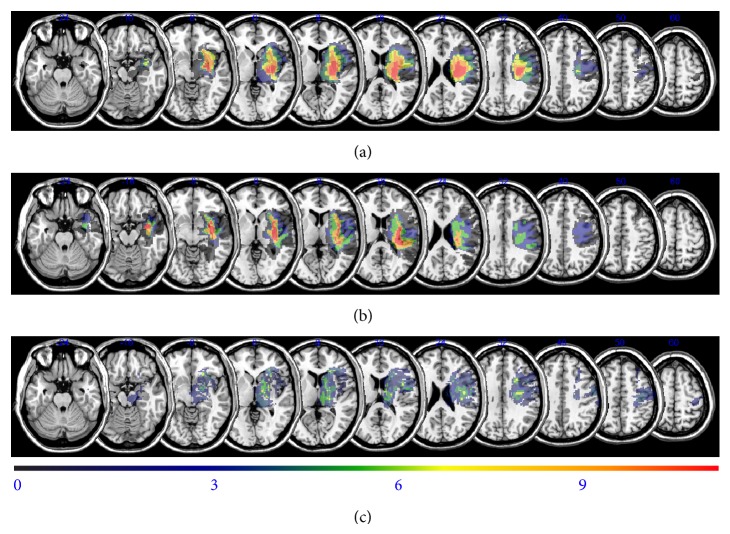
Overlay of lesions in the patients with hemorrhagic stroke. The top two figures represent the subject's upper limb motor function. The bottom shows the subtraction analysis in which the overlay of patients with isolated movement recovery was subtracted from the overlay of those with mass synergic movement recovery. (a) Overlay of lesions for patients with a synergic upper limb (*n* = 6). (b) Overlay of lesions for patients with an isolated upper limb (*n* = 5). (c) Subtraction analysis using (a) and (b).

The overlapping lesions of the brain included the putamen, thalamus, internal capsule, corona radiata, and primary and premotor cortices (Figure 1: the color represents the frequency of overlap). The lesions affecting upper limb function are shown in Figure 2, in which the subjects with selective motion (*n* = 5) and those with poor recovery and mass synergic motion (*n* = 6) are presented separately. The two groups did not differ in the size of the lesion, start of rehabilitation, or age. The data from the subtracted lesions indicate that poor recovery of the upper limbs was related to involvement of the internal capsule, anterior putamen, thalamus, periventricular white matter, and premotor cortex (Figure 2).

## 4. Discussion

Although previous studies have demonstrated that several brain lesions are related to the severity of functional outcome, the effects of specific brain lesions are not yet clear. This study investigated the effect of the stroke lesion on motor recovery in patients with hemorrhagic stroke. Our results suggest that involvement of the internal capsule, thalamus, periventricular white matter, and premotor cortex is related to poor upper limb recovery in patients with hemorrhagic stroke. The recovery of trunk control, lower limb motor function, and sensory function occurred rapidly within 3 months. Upper limb function and gait improved significantly from 3 to 6 months after the stroke.

Previously, we showed that motor function of the entire limb improved rapidly within 4 weeks but did not improve significantly from 3 to 6 months [8]. Focusing on hemorrhagic stroke, our results showed that recovery of the upper and lower limbs, sensory function, and gait improved until 3 months after onset. The recovery of gait improved over a longer period, until 6 months after onset. Another recent study obtained similar results, in which the functional improvement of the upper limb persisted up to 12 months after onset [15]. These results provide evidence of the effect of rehabilitation during the subacute period in stroke patients.

The relationship between functional status and specific brain lesions has long been investigated [3, 7, 16, 17]. The motor function of the upper limb, especially the hand, is more complex than that of the lower limb. This is a significant barrier in research on the effects of brain lesions on upper limb function. Previous research suggested that the involvement of the corona radiate and internal capsule was related to poor upper limb motor recovery [7]. Our result emphasizes the importance of the internal capsule and corona radiata in motor function in patients with stroke. However, the effects of other deep brain lesions on prognosis are not known. The anterior putamen and thalamus play roles in motor function [18]. The anterior putamen and thalamus are considered nonspecific motor areas [19]. The anterior putamen and thalamus are considered nonspecific motor areas [20]. According to our results, the anterior putamen and thalamus might play roles in the selective and isolated motor control of the upper limb. Our results support the role of the premotor cortex in the recovery of motor function, as previously reported in studies on monkeys [21].

Since our study had few subjects, we used several focusing methods to overcome bias. We recruited only patients with hemorrhagic stroke, supratentorial lesions, and moderate to severe hemiplegia. This allowed determination of the lesions that are important in upper limb motor function but did not reveal the changes associated with recovery or the connectivity among culprit lesions. Further research is needed to answer these remaining questions.

In conclusion, the involvement of the internal capsule, thalamus, periventricular white matter, and premotor cortex might be related to poor recovery of the upper limbs in patients with hemorrhagic stroke. These results are useful for planning rehabilitation strategies and understanding the prognosis of hemorrhagic stroke.

## Figures and Tables

**Table 1:** Baseline characteristics and lesion size of subjects.

Pt.	Paretic side	Sex	Age	Location/cause of lesion	Lesion volume (cm^3^)	Rehabilitation started (days after onset)
1	Rt. hemiplegia	M	53	ICH, BG, Lt.	18.13	24
2	Lt. hemiplegia	F	68	ICH, BG, Rt.	30.78	17
3	Rt. hemiplegia	F	26	ICH, T-P, Lt.	50.59	21
4	Rt. hemiplegia	F	46	ICH, BG, Lt.	12.99	10
5	Lt. hemiplegia	M	33	ICH, BG, Rt.	20.27	24
6	Lt. hemiplegia	M	53	ICH, thalamus, Rt.	19.16	17
7	Rt. hemiplegia	M	29	ICH, BG, Lt.	52.57	20
8	Lt. hemiplegia	M	52	ICH, BG, Rt.	52.09	11
9	Rt. hemiplegia	F	70	ICH, thalamus, Lt.	14.67	22
10	Lt. hemiplegia	M	43	ICH, BG, Rt.	58.66	10
11	Lt. hemiplegia	M	58	ICH, F-T, Rt.	49.66	13

			47.3 ± 15.1		34.51 ± 18.12	17.6 ± 5.6

BG, basal ganglia; ICH, intracerebral hemorrhage; Lt., left; Rt., right; SD, standard deviation; T-P, temporoparietal; F-T, frontotemporal.

Values are given in mean ± standard deviation.

**Table 2:** Clinical evaluations with time.

	The effect of time	Initial assessment	1 month after onset	Pairwise comparison (initial and 1 month)	3 months after onset	Pairwise comparison (1 and 3 months)	6 months after onset	Pairwise comparison (3 and 6 months)
TIS	<.001^*^	5.3 ± 5.3	11.6 ± 4.2	.005^†^	16.7 ± 3.7	.007^†^	18.0 ± 3.1	.027
Upper limb	<.001^*^	6.6 ± 6.2	10.0 ± 9.6	.039	23.2 ± 18.2	.003^†^	25.8 ± 18.8	.007^†^
Lower limb	<.001^*^	7.4 ± 4.4	10.7 ± 6.0	.017	18.3 ± 4.6	.003^†^	19.1 ± 4.0	.336
Sensory	<.001^*^	2.4 ± 4.6	4.0 ± 4.9	.066	11.6 ± 7.2	.003^†^	13.1 ± 8.1	.235
FAC	<.001^*^	0 (0-0)	0 (0-1)	.038	3 (3-4)	.003^†^	4 (3–5)	.007^†^

TIS, Trunk Impairment Scale, 0–23; upper limb, Fugl-Meyer Assessment arm section, 0–60; lower limb, Fugl-Meyer Assessment leg section, 0–28; sensory, Fugl-Meyer Assessment sensory section, 0–24; FAC, functional ambulation category, 0–5.

^*^ *P* value < 0.05 was deemed to be significant.

^†^ *Post hoc* analyses were conducted using a Bonferroni's correction (*P* < .0167).

The values of TIS, FMA arm, FMA leg, and sensory test are given in mean ± standard deviation.

The values of FAC are given in median (interquartile).
